# Weld Formation Mechanism and Microstructural Evolution of TC4/304 Stainless Steel Joint with Cu-Based Filler Wire and Preheating

**DOI:** 10.3390/ma12193071

**Published:** 2019-09-20

**Authors:** Junzhao Li, Yibo Liu, Zuyang Zhen, Peng Jin, Qingjie Sun, Jicai Feng

**Affiliations:** 1 State Key Laboratory of Advanced Welding and Joining, Harbin Institute of Technology, No.92 West Dazhi Street, Harbin 150001, China; 2 Shandong Provincial Key Laboratory of Special Welding Technology, Harbin Institute of Technology at Weihai, No.2 West Wenhua Road, Weihai 264209, China

**Keywords:** titanium alloys, stainless steel, cold metal transfer, intermetallic compounds, mechanical properties

## Abstract

Ti-Fe intermetallic compounds were effectively suppressed with Cu-based filler wire and weld formation was greatly improved with the preheating of substrates when joining TC4 titanium alloy and 304 stainless steel. A Ti/Cu transition zone consisting of complex TiCu, Ti_2_Cu_3_, TiFe, and TiFe_2_ phases was formed between Cu-weld/TC4 interface, while Cu-weld/304ss interface was mainly composed of α-Fe and ε-Cu solid solution. At lower heat input, the undercut defect in back surface had potential to cause crack initiation and joint fracture. Though increasing heat input would improve weld morphology, the formation of thick interfacial reaction layer and weld cracking led to low weld quality and joint strength. The preheating of substrates had an obvious effect on wetting ability of liquid filler metal and could achieve a better weld quality at lower heat input. The back formation of weld was improved to decrease the occurrence of weld defects. The highest tensile strength of 365 MPa occurred at welding heat input of 0.483 kJ/cm, increasing by 47% compared to the joint without preheating. The interfacial reaction mechanism was discussed to reveal the relationship between microstructural characteristics and fracture behavior of Ti/steel welded joints with Cu-based filler wire.

## 1. Introduction

Titanium-steel dissimilar junctions are increasingly applied in chemical, power generation, and spacecraft industries due to their excellent corrosion resistance, lower cost, and weight. The main difficulty in joining titanium alloy with steel using fusion welding was the massive formation of brittle FeTi and Fe_2_Ti intermetallic phases [1]. It was confirmed that the type, amount, and distribution of the intermetallic phases greatly affected the mechanical behavior. These highly brittle intermetallic phases were considered as the source of crack and caused joint fracture with lower mechanical properties [2].

Solid-state process, such as diffusion bonding, friction stir welding and explosive welding, are effective in controlling the formation of brittle intermetallic phases, but the application of these methods were restricted by structure configurations. Multiple reaction layers of Ti-Fe phases formed at the interface during welding [3]. Dey et al. [4] studied that the direct friction welding of Ti to 304L. SS had a stronger weld and tensile failure occurred in the Ti base material. Fusion welding methods have great flexibility in weld configuration, high welding productivity, and are widely used in joining titanium alloys and steel. However, it is difficult to obtain an admirable dissimilar joint when directly fusion welding titanium alloy and steel, because the formation of brittle Ti-Fe phases would immediately lead to joint crack. Chen et al. [5] reported the tensile strength of the joint was higher when the laser beam was offset toward the stainless steel than toward the titanium alloy because of the suppression of melting of TC4 plate, and the highest value was 150 MPa.

Previous research has indicated that the intermediate layers can effectively modify the phase compositions and suppress the reaction between Ti and Fe. Cu interlayer could limit the diffusion of Ti into the melted zone and no considerable accumulation of TiFe_2_ took place at the interface between the melted zone and steel [6]. Vanadium element exhibited good bonding with adjacent materials in Ti-Ti and Fe-Fe welded joints [7]. Atom diffusion and migration between Ti and Fe or C were effectively prevented by adding pure Ni as the interlayer metal, and a firm joint was obtained [8]. Therefore, an intermediate layer can limit the atom diffusion to suppress the formation of brittle IMCs and form some other IMCs. Tomashchuk et al. [9] described that the beam offset greatly affected the mechanical properties. They reported electron beam shift toward the steel side and Cu interlayer could inhibit the melting of titanium alloys and the formation of brittle Fe-Ti phases. The maximum tensile strength of 350 MPa was achieved with 40-80 μm diffusion path length of Ti. Hao et al. [10] found a Ti/Cu reaction zone formed between TC4 substrate and the weld using copper-based filler wire, FeTi and Fe_2_Ti compounds also produced with increasing welding heat input. However, all the joints fractured at the Ti/Cu reaction zone due to brittleness of Ti-Cu phases in nature. According to the Ti-Cu and Cu-Fe binary phase diagram, various Ti/Cu brittle phases formed at the diffusion transition zone, however the microstructure of the Cu-Steel interface was mostly solid solution phases. Yao et al. [11] claimed that the solid solution phases have greatly properties than brittle intermetallic phases. Cao et al. [12] suggested that an excellent Ti/Cu joint can be achieved by CMT method and all the joints fractured in the heat affected zone of Cu with plastic fracture mode. This indicated that a layer of intermetallic compounds with 120 μm was controlled with different lap configuration. Zhao et al. [13] laser welded titanium with copper and found that a thin intermetallic layer about 25 μm was formed between weld metal and Ti substrate with laser offset to copper. Intermetallic compounds such as TiCu, TiCu_2_, Ti_2_Cu and Ti_3_Cu_4_, were formed and considered as the weakest zone of the joint. Chen et al. [5] investigated copper-steel welded joints with laser welding and the results indicated that the tensile strength was weakly dependent on melting of the copper. Magnabosco et al. [14] joined copper plate with three different stainless steel plates and showed complex heterogeneous fusion zone microstructures characterized both by rapid cooling and poor mixing of the materials. The transition zone contained main elements which were mutually insoluble. Besides, the joint geometry can increase effective bonding area and greatly improve the weld quality. The wettability and spreading behavior of molten metal played an important role on morphology of the welded joint. Pulsed double electrode gas metal arc to weld aluminum and galvanized mild steel, and found that the droplet alternation transfer could improve wettability of molten aluminum [15]. The magnetic field to affect the flowability and surface spreadability of the molten filler metal on TA2 plate, thus improved the wetting behavior [16]. The effects of heat input on the microstructure of Al/Cu joint using a low heat input pulsed double-electrode gas arc welding [17]. A quadratic relation between the thickness of the IMC layer and welding heat input was derived using the theory of thermal activation process. Besides, the modified droplet transfer process may affect weld formation process. Cao et al. [18] investigated the effect of Zn-coating on the wetting process of liquid droplets and found that the evaporation of Zn could decrease the temperature at the interface and make the steel to expose the fresh metallic surface which was much easier to be spread by a liquid droplet.

In this study, TC4 titanium and 304 stainless steel joints are fusion welded by cold metal transfer (CMT) using S201 Cu-based filler wire. Firstly, the wettability of Cu filler wire on TC4 alloy and 304 stainless steel was investigated by high speed camera during CMT welding process. Then, butt joining experiments of TC4 alloys with 304 stainless steel are conducted to study the effect of welding heat input on weld formation, microstructure, and mechanical properties. Finally, preheating is adopted to further improve joint quality.

## 2. Materials and Methods 

**Figure 1:**
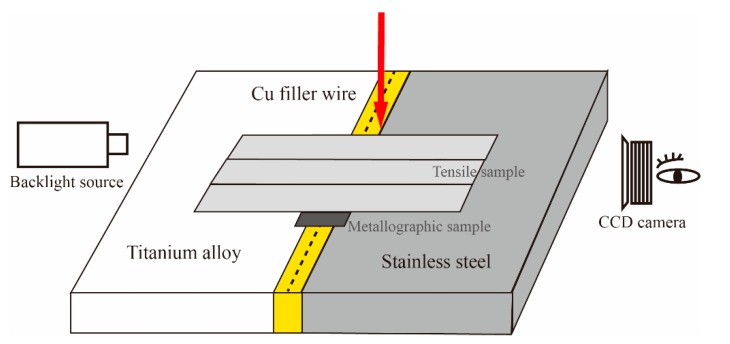
The welding configuration.

Butt joining of TC4 titanium alloy to 304 stainless steel with thickness of 1 mm was conducted by cold metal transfer process. The welding configuration was illustrated in Figure 1. Prior to the welding process a gap of 0.5 mm was presented between the TC4 alloy and 304 stainless steel substrates by feeler gauge to ensure the butt gap width. A suitable weld gap enabled a high-quality welded joint at a smaller welding heat input, and meanwhile could avoid the formation of cracking. The heating device was used to control preheating temperature and the heating panel was placed on the back of the substrates. The wetting experiments of Cu filler wire on TC4 alloy and 304 stainless steel were observed by high-speed video camera (i-SPEED3, Olympus, Tokyo, Japan) with a frame rate of 2000 frames per second. The background laser beam was projected towards the wire/droplets when collecting the metal transfer process. Spot welding method was adopted with the static welding torch, and then the high-speed camera recorded the whole metal transfer process. Tomashchuk et al. [9] suggested the beam offset to steel side suppressed the formation of brittle IMCs, so the arc was pointed to the edge of 304 stainless steel to decrease the fusion of TC4 titanium alloy and increase wetting on 304 stainless steel plate in this study. S201 copper filler wire with 1.2 mm diameter was used. The chemical compositions of base metals and filler wire are shown in Table 1. The two plates were polished by stainless steel wire brush and then cleaned with acetone. The welding process was performed using TransPuls Synergic (TPS) Advanced 4000 type CMT welding system, and a pure CMT mode was selected. The welding parameters and heat input are listed in Table 2, which is calculated by Q = ηUI/v, where U, I, v and η are welding voltage, welding current, welding speed, and welding process efficiency, respectively. The CMT welding process with the controlled dip transfer had a process efficiency of ~85% when measured with the liquid nitrogen calorimeter [19].

After welding, the joints were perpendicularly cross-sectioned to the welding direction for metallographic analysis. Backscattered electron image observation and quantitative compositions identification of the interfacial microstructure were conducted by scanning electron microscope (SEM, MERLIN Compact, Zeiss, Heidenheim, Germany) equipped with an energy-dispersive X-ray spectrometer (EDS). Standard tensile coupons (Instron 5967, Instron, Boston, MA, USA) were performed at a constant speed of 1.0 mm/min (stain rate of 4.2 × e^−4^ s^−1^) for the purpose of determining the cross-weld tensile properties. The fractured surfaces of tensile specimens were observed by SEM.

## 3. Results

### 3.1. Macro-Morphology

**Figure 2:**
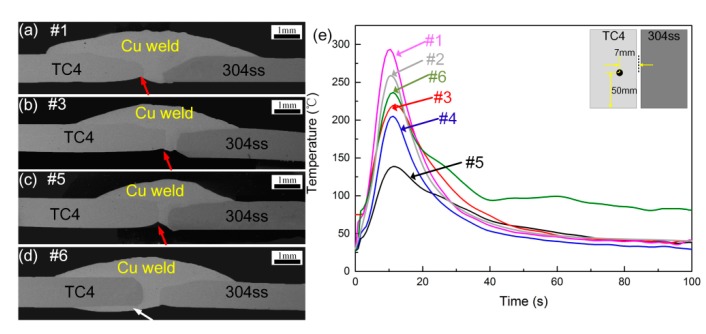
Macrophotogragh of the cross section of the hybrid joint (**a**) #1; (**b**) #3; (**c**) #5; (**d**) #6; (**e**) thermal cycles under different welding conditions.

The cross sections of TC4 titanium alloys and 304 stainless steel butt joints produced by various welding parameters are exhibited in Figure 2. It can be seen that various welding currents and speeds were used to join these two materials, however, welding deformation appeared with unmatched parameters due to the different thermal physical properties among titanium alloys, stainless steel, and copper filler wire. The various gap width of welded joint was mainly caused by the deformation degrees and melting amounts of parent metals in different welding heat input. Figure 2a–c show that with increase of welding heat input, the spreading ability of liquid Cu filler wire increased and some pores can be observed in the transition zone due to the fierce metallurgical reaction. The 304ss plate was melted with increasing welding heat input due to the offset of arc, forming a mixed Cu-steel interface. TC4 plate was slightly melted and reacted with the liquid molten Cu filler wire to form a Cu-Ti interface. The dilution ratio of TC4 alloy in the weld was increased as heat input improved. A clear undercut can be seen at the Cu-TC4 back interface. This is caused by the offset of arc and a fast solidification of the melt pool when in contact with the back of TC4 plate. The undercut defects (red arrow in Figure 2) in the back of weld may cause stress concentration in the process of tensile test, increasing the tendency of crack initiation. Increasing welding heat input can improve the back formation of weld, however the higher heat input may result in large melting of TC4 plate and form thick and brittle transition layer. This also had an adverse effect on weld mechanical properties. It can be seen from Figure 2d that the preheating of substrates can increase the spreading ability of liquid Cu filler wire on TC4 surface at a lower heat input (white arrow in Figure 2). The wettability of Cu filler metal in both the back surface was improved and the molten Cu-filler wire can form wraparound effect on TC4 plate, increasing the effective bonding areas of Ti-Cu interface.

To better clarify the wetting behavior at the Cu-weld/TC4 interface under different welding conditions, the thermal cycles at specific spots (50 mm distance from welding started point and 7 mm distance from welding center) detected by thermocouple and wetting process were shown in Figure 2e. It can be seen that the peak temperature of TC4 measured plot gradually decreased with decrease of heat input, causing the wetting of Cu-based filler wire on TC4 plate constrained. The preheating of substrate can slightly increase peak temperature to improve wetting of filler metal on TC4 plate as seen in Figure 2e. The microstructural evolution of weld joint is unlikely to change, however the preheating of substrate can improve the back formation of welded joint. Low welding heat input with preheating was used to promote wetting ability of molten metal, decrease the thickness of IMCs layer and welding deformation.

**Figure 3:**
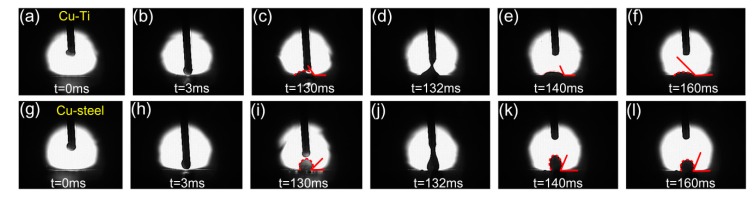
The wetting process of Cu filler wire on TC4 alloy and 304 stainless steel. (**a**) t = 0 ms; (**b**) t = 3 ms; (**c**) t = 130 ms; (**d**) t = 132 ms; (**e**) t = 140 ms; (**f**) t = 160 ms; (**g**) t = 0 ms; (**h**) t = 3 ms; (**i**) t = 130 ms; (**j**) t = 132 ms; (**k**) t = 140 ms and (**l**) t = 160 ms.

**Figure 4:**
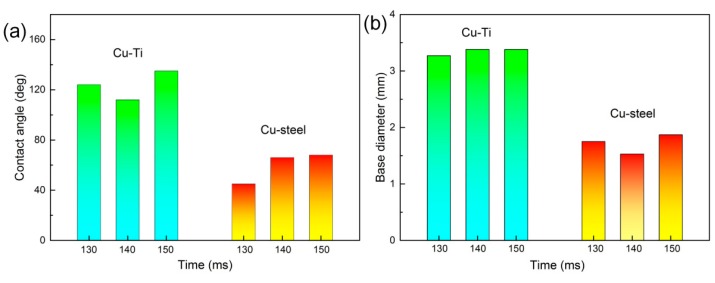
Variation in contact angles and base diameter with time.

Furthermore, the wetting behavior of molten Cu filler wire on TC4 and 304 stainless steel substrates during the cold metal welding process was observed to reveal the weld formation. The variation of contact angles and base diameter with time for the molten droplet on substrates was shown in Figure 3 and Figure 4. It is worthy to note that almost all contact angles and base diameters on TC4 plate are larger than that on 304 stainless steel plate, indicating the better wettability on TC4 plate. The preheating of the substrates can promote the wetting ability of liquid filler wire. Therefore, the arc can be pointed to the edge of 304 stainless steel to decrease the fusion of TC4 titanium alloy, the lower welding heat input can be adopted to suppress the formation of brittle intermetallic phases and improve wetting with preheating.

The different wetting behavior of Cu/Ti and Cu/Fe plates can be attributed to the various reactive wetting mechanisms. Wetting is not only influenced by the interactions with the welding heat input, but also by the liquid-solid interaction occurring at the interface. Differences in the interfacial reaction modify the wetting kinetics. Cu has a high reactivity to Ti and various Cu-Ti reaction products formed at Cu/TC4 interface. Lin et al. [20] suggested that such intermetallic phases always have a relatively large volume per mole, which may be a factor for promoting the wettability. However, α-Fe and ε-Cu solid solution are presented at Cu/304ss interface due to immiscibility. Bernardo et al. [21] reported the dissolution effects appear to slow down the spreading rate and lead to a poor wetting. Furthermore, heat conductivity coefficient of 304 stainless steel is much larger than TC4 titanium alloys, indicating rapid solidification of liquid Cu weld occurs on 304ss plate and wetting evolution is no longer possible. So the wettability of molten Cu filler wire on TC4 plate was more obvious than that on 304ss plate.

### 3.2. Microstructural Analysis

**Figure 5:**
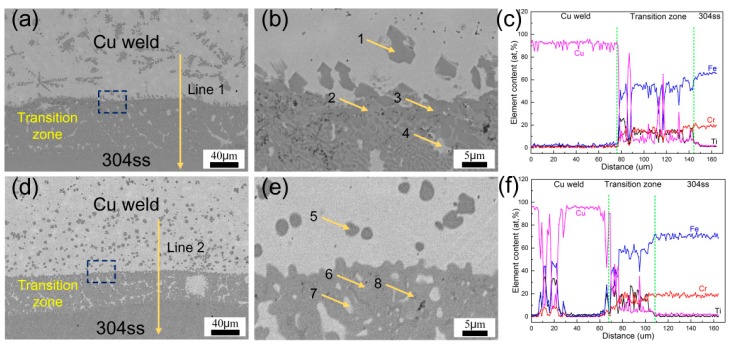
SEM images and line scanning of the steel/weld interface. (**a**–**c**) #1; (**d**–**f**) #3.

The Cu-weld/304ss interfaces of samples #1 and #3 were shown in Figure 5, and the EDS results of different phases were shown in Table 3. The results show that at high heat input of 0.625 kJ/cm a 70 μm wide transition zone between Cu-weld/304ss interface formed due to more dissolving of 304 stainless steel. The higher welding heat input also promoted the dissolution of TC4 base metal and some Ti content migrated in the Cu-weld/304ss side. Some blocky phases distributed in transition zone near to the Cu-weld. The EDS results indicated that the transition zone mainly consisted of Fe, Cu, Cr, Si, and less Ti, and the Ti content gradually decreased from Cu-weld to 304ss side. Fewer TiFe_2_ and TiFe IMCs (dark phases) were formed in transition zone near to the Cu weld due to the largely melting of TC4 plate and fluid flow. However, with decrease of welding heat input, the thickness of transition zone between Cu-weld/304ss interface was decreased to 40 μm due to the slightly melting of base metal. The reaction of Ti-Fe was restrained because of the decrease of Ti content in the Cu-steel interface. At lower heat input of 0.483 kJ/cm the Cu weld/304ss interface mainly consisted of ε-Cu and α-Fe solution with fewer TiFe_2_ phases.

**Figure 6:**
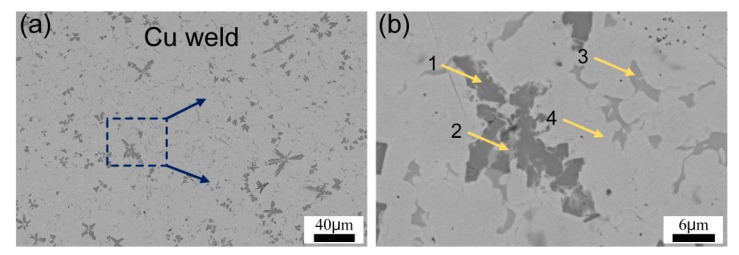
(**a**) Microstructure in weld zone of sample #3; (**b**) the enlarged image of the box in (**a**).

The microstructure of Cu-weld zone in sample #3 is presented in Figure 6 and the EDS analysis results are shown in Table 4. The matrix was ε-Cu solid solution, the dark flower-like phases were TiFe, TiFe_2_ and Ti_5_Si_3_ IMCs, and the gray irregular phase was Ti_2_Cu_3_ distributing in ε-Cu solid solution matrix. This is because partial dissolution of TC4 and 304ss substrates promoted the diffusing of Ti and Fe atoms into Cu-weld. Besides, the Si atom was segregated in the IMCs from the EDS results. Lin et al. [22] suggested that the adsorption energy based on the affinities can be used for the explanation of interfacial structure, and it can be expressed as following,

(1) $$E_{M(B)}^{\infty \mathrm{SL}}{=m}_{1}{(\lambda }_{\mathrm{AM}}{-\lambda }_{\mathrm{BM}}{-\lambda }_{\mathrm{AB}})$$

The chemical adsorption was usually considered as a prerequisite for the precipitation of reaction product when the concentration of the adsorbate was below the saturation adsorption concentration. Therefore, $E_{M(B)}^{\infty \mathrm{SL}}$/m_1_ can be used for the prediction of interfacial structure before experiment. $E_{M(B)}^{\infty \mathrm{SL}}$/m_1_ for Si-Cu/Ti system was −122.5 KJ/mol, for Si-Cu/Fe system was −85.5 KJ/mol. The negative value suggested that Si can segregate at IMCs layer.

**Figure 7:**
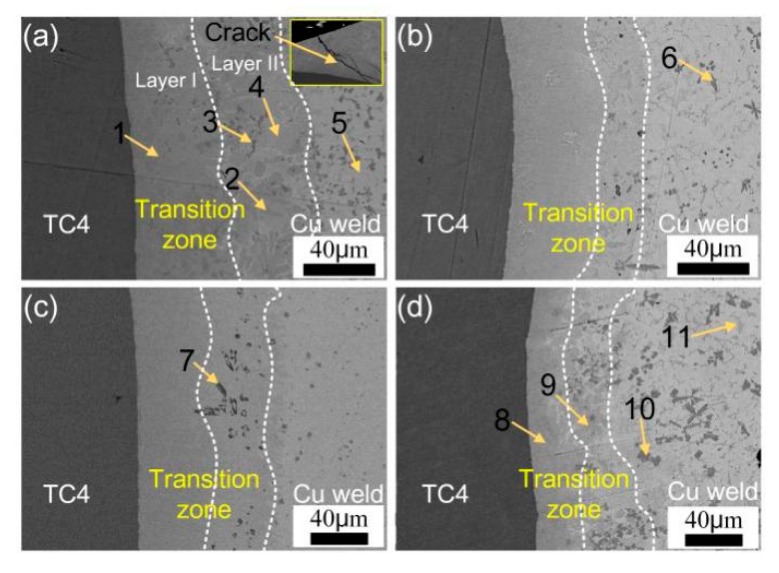
Microstructure of the TC4/Cu-weld interface. (**a**) #1; (**b**) #3; (**c**) #5; (**d**) #6.

Figure 7 shows the microstructure of the TC4/Cu-weld interface with different welding heat input. TC4 titanium alloy was slightly melted at lower welding heat input, however with increase of heat input the melting of TC4 plate increased and more Ti was dissolved into reaction zone. An irregular reaction layer was formed between TC4/Cu-weld zone. It can be seen that the TC4/Cu-weld transition layer was divided into two layers, a continuous reaction layer and a mixed reaction zone. A large amount of grey phases distributed in the entire TC4/Cu-weld transition zone and some dark flower-shaped phases located among the grey matrix near to the Cu-weld zone. Furthermore, the Ti content was decreased gradually from TC4 plate to Cu-weld, resulting in the formation of different IMCs phases. The thickness of TC4/Cu-weld transition zone decreased from 105 μm to 80 μm with decrease of heat input. Combination with EDS analysis results, the transition zone mainly consisted of continuous TiCu phase near to the TC4 plate, and some TiFe, TiFe_2_ and Ti_2_Cu_3_ phases were dispersedly distributed in the weld/TC4 interface. However, the amount of TiFe decreased with decrease of heat input. At a higher heat input of 0.625 kJ/cm, the Cu-weld/TC4 reaction layer was composed of a continuous layer and a dispersed layer. As indicated in Table 5, the Ti content in layer II was lower than that in layer I. Some weld cracking was also observed at the weld toe area due to the brittleness and weld deformation as seen from Figure 7a. The thickness of reaction layer was decreased with the decrease of welding heat input. Furthermore, the preheating of substrates could improve metal fluid and the wetting ability of liquid weld metal, forming a thin transition zone at lower heat input. The TC4/Cu-weld interface was mainly composed of TiCu phase layer and a complex Ti-Cu, Ti-Fe maxed layer with 80 μm thickness, as shown in Figure 7d. The thickness of continuous layer was obviously decreased with the preheating.

**Figure 8:**
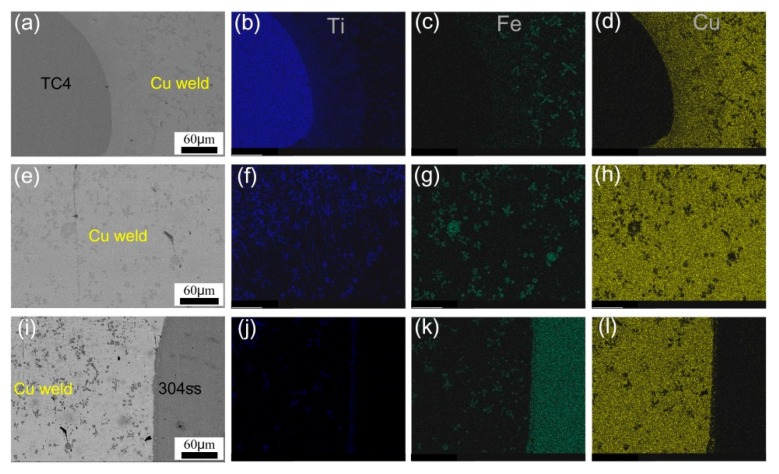
EDS element distribution maps in different zones of sample 6. (**a**–**d**) TC4 side; (**e**–**h**) weld zone; (**i**–**l**) 304 stainless steel side.

The distribution of alloying elements Ti, Cu, and Fe in the different welded zone of sample #6 is shown in Figure 8. It can be seen that Ti gradually decreased while Fe increased from TC4 to Cu weld and to 304ss substrate, indicating the Fe, Ti, and Cu elements were diffused and reacted with each other in the welded joint. And this phenomenon was aggravated with increase of welding heat input. The substrates of sample #6 were slightly melted due to a lower heat input, so a small quantity of Fe and Ti were gathered in the reaction zone.

The interfacial reaction mechanism and microstructural evolution are described as follows. As the formation of intermetallic phases is dependent of diffusion and reaction between the substrates, an increase in time-temperature cycles increases the mobility of metals and, consequently, forming various phases. The peak temperature was gradually decreased from 300 °C to 130 °C with decrease of heat input, as shown in Figure 2. The decrease of interfacial temperature restrained the dissolution and diffusion of TC4 plate, indicating that the formation of Ti-Cu phases was suppressed. The thermal cycle results were corresponded with the microstructural evolution at the Cu-weld/TC4 interface. The preheating of substrate slightly increased the peak temperature of the interface and decreased the cooling speed above 100 °C, thus the microstructure of the Cu-weld/TC4 interface was nearly unchanged. The decreased cooling rate could reduce the tendency to generate welding cracking and increase wetting process, which may improve joint quality.

**Figure 9:**
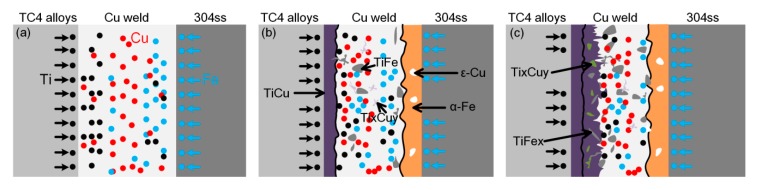
Schematic of element diffusion and microstructure evolution. (**a**) atoms diffusion process; (**b**) the subsequent solidification process and (**c**) the finial microstructure in weld.

Figure 9 presents the microstructural evolution of Cu-weld/TC4 and Cu-weld/304ss interface. The 304 stainless steel was largely melted and formed a mixed transition zone with Cu filler wire at high welding heat input. The Cu and Fe alloying elements diffused and infiltrated to each other due to infinite miscibility of these two elements in liquid phases. However, as the solubility of Fe in Cu is much lower than the solubility of Cu in Fe, and the solubility decreased with decrease of temperature, some spherical Fe particles dispersed in Cu-weld zone and some Cu solution gathered in the Fe solid solution. The TC4 plate was also melted at higher heat input and some Ti element diffused to the Cu-weld/304ss interface, forming brittle Fe-Ti phases. So the Cu-weld/304ss interface was mainly composed of α-Fe, ε-Cu solution, less Ti-Fe and Ti-Cu brittle phases. At higher welding heat input, a Cu-weld/TC4 interface layer consisted of complex Ti-Cu phases TiCu, TiCu_2_, Ti_2_Cu_3_, TiFe_2_ and TiFe due to diffusion of elements. The molten Fe was diffused and reacted with Ti alloying element. Besides, from the microstructure image, some cracks formed due to large deformation and pores defects also occurred in the welded joint. The thermal expansion coefficient and thermal conductivity of copper were significantly higher than those of the steel [23]. Hence, during the welding process, the large misfit strain and the residual stresses will be inevitably generated in the joint, leading to the solidification cracking. The porosity was a common defect originating from hydrogen which was highly soluble in liquid copper. The combination effect of thick brittle phases and weld cracks in the Cu-weld/TC4 interface is favorable to crack initiation, which causing a low joint mechanical properties. At lower heat input, the steel substrate was slightly melted and formed a flat and narrow transition layer. Due to the melting and diffusion of Fe element, the Cu-weld/304ss microstructure was mainly ε-Cu solid solution, accompanied by the dispersion distribution of α-Fe solid solution. The TC4 alloy substrate was also slightly melted under the effect of thermal cycle. The liquid molten Cu filler wire was wetting and spreading on the TC4 alloy surface, forming a thin reaction layer between TC4 substrate and weld metal. Only some Ti-Cu intermetallic phases and Ti-Fe phases formed at the TC4/weld interface. The Ti alloying element diffused into weld zone and reaction with Cu filler wire, it can be observed that some Ti_2_Cu phases were distributed in the weld zone adjacent TC4/weld interface. A small amount of Ti-Fe IMCs distributed in the Cu weld zone due to the dissolution of 304ss and fluid flow. The less brittle phases and increased bonding area are beneficial to achieve a high tensile strength.

Under the same welding heat input, the thermal cycle results presented that the temperature on the reaction interface was similar at the same heat input, indicating the phases and thickness of interfacial reaction layer had a strong resemblance. However, from the wetting behavior and weld appearance, the preheating could largely promote the back formation of TC4 substrate side and increase the effective bonding area of welded joint. The joint with preheating had a great tensile strength than that without preheating, showing that the weld formation can largely affect the TC4/304ss welded joint quality.

### 3.3. Tensile Test and Fractography

**Figure 10:**
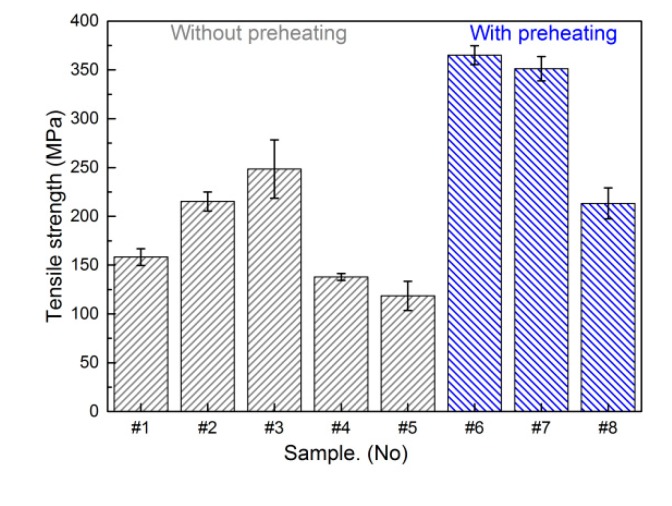
Tensile fracture load of TC4 titanium alloy/304 stainless steel butt joint.

The effects of welding heat input and preheating temperature on tensile strength of the joint were studied and the tensile test results are presented in Figure 10. The tensile fracture load increased first and then decreased with decrease of heat input. However, the values of tensile load remained low and the joint fractured at Cu-weld/TC4 interface for a high welding heat input conditions, which corresponded to a brittle fracture. The highest value of load of 248 MPa was obtained for the heat input 0.483 kJ/cm. Pardal et al. [24] found that the tensile result was 200 MPa using a CuSi-3 welding wire and the interfacial failure could not be avoided. The preheating of substrates had great effect on joint strength and the joint tensile fracture load increased by 47% compared to the joint without preheating. The maximum tensile strength of welded joint with preheating can reach to 365 MPa. It indicated that the wetting behavior of Cu-based filler wire on TC4 and 304ss substrates played an important role on joint strength and the fracture mechanism of welded joint was discussed as followed.

The fracture path and surface were studied by SEM-EDS in order to correlate the weld microstructure with mechanical properties. The joints fractured at Cu-weld/TC4 reaction zone at high heat input, meaning the thick Ti-Cu and Ti-Fe brittle reaction layer were the weakest zone in butt joints. Besides, some weld defects such undercut, cracking and pores were formed at this zone, favoring the origin of cracks and causing a low tensile property. With lower welding heat input due to less diffusion of Ti and Fe atoms the TC4/Cu-weld side consisted of ε-Cu, TiCu phases and TiFe phases with thin thickness, however the undesirable weld morphology with small bonding area led to a poor joint quality. The preheating of substrates can increase the effective bonding area of welded joint, especially the back formation of welded joint was improved. The excellent weld formation was beneficial to prevent the generation of cracks. Therefore, the joint mainly fractured at TC4/Cu-weld with a relatively higher tensile strength.

**Figure 11:**
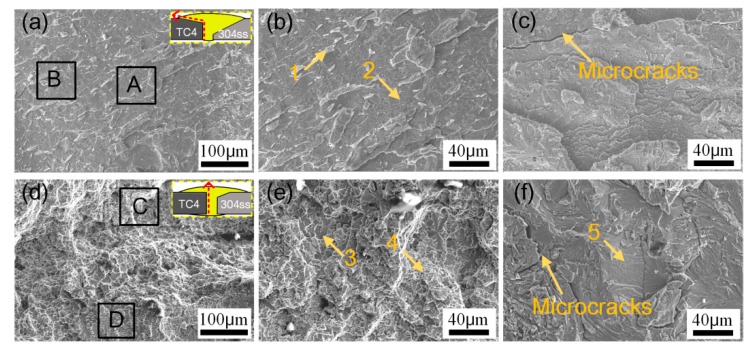
Fracture paths and morphologies of joints. (**a**–**c**) in sample #1; (**d**–**f**) in sample #6.

The fracture paths and surfaces of sample #1 and sample #6 were observed by SEM with EDS analysis, as shown in Figure 11 and Table 6. At higher heat input of 0.625 kJ/cm in sample #1, the joint fractured along Cu-weld/TC4 interface. Fracture surface showed flat cleavage fracture characteristics and obvious river-like pattern with some microcracks, shown in Figure 11a–c. The high heat input promoted form a thick and brittle interfacial layer. Besides, large deformation was induced and the microcracks were formed at the root of the joint as shown in Figure 7a. Hao et al. [10] reported the similar fracture surface feature of Ti-steel joint. However, Figure 11d–f presented that the fracture surface produced at low heat input of 0.483 kJ/cm in sample #6 with preheating exhibited a mixed fracture mode. The joint was fractured along the interfacial IMCs layer and Cu weld. According to the EDS results, the fracture surface was mainly composed of Ti-Cu phases.

## 4. Conclusions

In this study, the microstructure and mechanical properties were examined to reveal the effect of Cu-based filler wire and preheating on joining TC4 and 304 stainless steel via cold metal transfer process. The main conclusions are listed as follows:

(1) It was found that Cu exhibited good bonding behavior with Ti than Fe. Cu-Ti weld metal was characterized by intermetallic phases, while Cu-Fe weld metal was presented by solid solution. The wettability of Cu-based filler metal on substrates was increased with preheating, which improved the back formation of weld and suppressed undercut defects.
(2) Using Cu-based filler wire was feasible to join TC4 titanium alloy and 304 stainless steel. The tensile fracture load increased with decrease of heat input to 0.483 kJ/cm, while decreased with further lower heat input due to incomplete bonding. The preheating can effectively improve joint tensile strength and the highest tensile fracture load of 365 MPa increased by 47% compared to the joint without preheating.
(3) The Cu-weld/304ss interface consisted of α-Fe, ε-Cu solution and fewer Ti-Cu phases due to diffusion of atoms. The Cu-weld zone was mainly composed of Ti-Cu phases distributed at ε-Cu matrix. Cu-weld/TC4 reaction zone consisted of thick Ti-Cu reaction layer and some Ti-Fe phases, and its thickness decreased with decrease of heat input because of less diffusion of Fe content into this zone. The brittle phases at Cu-weld/TC4 reaction layer led to deterioration of mechanical properties of Ti/steel joint.
(4) The joints fractured at the Cu-weld/TC4 interface at high welding heat input, revealing that the Ti-Cu and Ti-Fe reaction zone was the weakest location of the joint. The weld defects such as cracking, pores, and undercut served as the crack initiation, resulting in a poor joint strength. However, a lower heat input with preheating increased the effective bonding area and less brittle phases suppressed crack extension.

## Figures and Tables

**Table 1:** The chemical compositions of base metals and filler wire.

Plate	Ti	Al	V	Fe	C	Cr	Cu	Sn	Mn	Si	Ni
TC4	Bal.	5.8	3.6	0.3	0.1	-	-	-	-	-	-
304ss	-	-	-	Bal.	0.04	18.3	0.18	-	1.09	0.6	8.01
S201	-	-	-	-	-	-	Bal.	0.7	0.4	0.3	-

**Table 2:** The welding parameters and conditions.

Sample (No)	Welding Current (A)	Welding Voltage (V)	Welding Speed (mm/s)	Heat Input (kJ/cm)	Preheating Temperature (°C)
#1	70	10.5	10	0.625	-
#2	65	10.4	10	0.575	-
#3	75	10.6	14	0.483	-
#4	70	10.5	14	0.446	-
#5	65	10.4	14	0.411	-
#6	75	10.6	14	0.483	75
#7	70	10.5	14	0.446	75
#8	65	10.4	14	0.411	75

**Table 3:** EDS results of the phases in different locations in Figure 5 (at.%).

Position	Ti	Cu	Fe	Cr	Si	Possible Phases
1	25.4	4.3	54.2	10.5	5.6	α-Fe + TiFe_2_
2	10.1	16.1	56.7	11.2	2.2	α-Fe + ε-Cu + TiFe_2_
3	23.3	4.4	53.3	11.5	7.5	α-Fe + TiFe
4	2.1	86.4	8.4	2.0	1.1	ε-Cu
5	2.0	7.1	69.8	17.2	3.9	α-Fe
6	2.2	7.7	73.7	15.7	0.7	α-Fe
7	1.9	87.6	6.9	2.2	1.4	ε-Cu
8	23.8	4.1	59.3	12.1	0.7	α-Fe + TiFe

**Table 4:** EDS results of the phases in Cu weld zone in Figure 6 (at.%).

Position	Ti	Cu	Fe	Cr	Si	Possible phases
1	34.2	8.9	34.9	6.6	15.4	TiFe_2_ + Ti_5_Si_3_
2	48.9	25.0	14.5	2.1	9.5	TiFe_2_ + TiFe + ε-Cu
3	36.5	49.6	6.4	1.9	5.6	Ti_2_Cu_3_
4	4.2	95.8	-	-	-	ε-Cu

**Table 5:** EDS results of the phases in different locations in Figure 7 (at.%).

Position	Ti	Cu	Fe	Cr	Si	Al	Possible Phases
1	43.2	40.1	0.8	4.2	2.4	9.3	TiCu
2	26.0	56.0	5.2	1.3	4.1	7.4	Ti_2_Cu_3_
3	24.2	2.5	56.3	6.6	5.0	5.4	TiFe_2_
4	25.3	58.6	2.5	1.9	6.2	5.5	ε-Cu + TiCu
5	8.5	84.3	0.8	2.9	2.5	1.0	ε-Cu
6	31.0	5.2	44.4	5.2	8.6	5.6	TiFe + TiFe_2_
7	25.5	16.3	34.5	2.6	14.2	6.9	TiCu + TiFe_2_
8	45.6	44.2	0.5	0.8	2.6	6.3	TiCu
9	28.6	55.4	4.5	1.2	4.2	6.1	Ti_2_Cu_3_
10	32.8	6.3	47.6	6.3	1.8	5.2	TiFe + TiFe_2_
11	17.6	75.6	2.4	1.2	2.3	0.9	ε-Cu + TiCu_4_

**Table 6:** EDS results of the phases in different fracture locations in Figure 11 (at.%).

Position	Ti	Cu	Fe	Cr	Si	Possible Phases
1	36.5	56.9	2.6	3.7	0.3	Ti_2_Cu_3_
2	38.5	50.5	2.1	8.5	0.4	TiCu
3	27.8	69.5	1.3	1.1	0.3	ε-Cu + TiCu
4	21.8	74.7	3.2	0.2	0.1	ε-Cu + TiCu
5	53.7	38.6	2.4	4.8	0.5	Ti_2_Cu
